# Artificial surface labelling of *Escherichia coli* with StrepTagII antigen to study how monoclonal antibodies drive complement-mediated killing

**DOI:** 10.1038/s41598-023-46026-x

**Published:** 2023-11-01

**Authors:** Remy M. Muts, Maurits A. den Boer, Bart W. Bardoel, Piet C. Aerts, Carla J. C. de Haas, Albert J. R. Heck, Suzan H. M. Rooijakkers, Dani A. C. Heesterbeek

**Affiliations:** 1 Department of Medical Microbiology, University Medical Center Utrecht, 3584 CX Utrecht, The Netherlands; 2 Biomolecular Mass Spectrometry and Proteomics, Bijvoet Center for Biomolecular Research and Utrecht Institute of Pharmaceutical Sciences, Utrecht University, 3584 CH Utrecht, The Netherlands; 3 Netherlands Proteomic Center, 3584 CH Utrecht, The Netherlands

**Keywords:** Immunological techniques, Antibody isolation and purification, Microbiology techniques, Bacterial infection, Complement cascade

## Abstract

Antibodies play a key role in the immune defence against Gram-negative bacteria. After binding to bacterial surface antigens, IgG and IgM can activate the complement system and trigger formation of lytic membrane attack complex (MAC) pores. Molecular studies to compare functional activity of antibodies on bacteria are hampered by the limited availability of well-defined antibodies against bacterial surface antigens. Therefore, we genetically engineered *E. coli* by expressing the StrepTagII antigen into outer membrane protein X (OmpX) and validated that these engineered bacteria were recognised by anti-StrepTagII antibodies. We then combined this antigen–antibody system with a purified complement assay to avoid interference of serum components and directly compare MAC-mediated bacterial killing via IgG1 and pentameric IgM. While both IgG1 and IgM could induce MAC-mediated killing, we show that IgM has an increased capacity to induce complement-mediated killing of *E. coli* compared to IgG1. While Fc mutations that enhance IgG clustering after target binding could not improve MAC formation, mutations that cause formation of pre-assembled IgG hexamers enhanced the complement activating capacity of IgG1. Altogether, we here present a system to study antibody-dependent complement activation on *E. coli* and show IgM’s enhanced capacity over IgG to induce complement-mediated lysis of *E. coli*.

## Introduction

Antibodies play a key role in the immune defence against Gram-negative bacterial infections. Gram-negative bacteria are notoriously difficult to combat because of their complex cell envelope structure^1^. This cell envelope structure consists of an inner membrane, a peptidoglycan layer, and on top an outer membrane decorated with outer membrane proteins and lipopolysaccharides (LPS)^2^. The broad diversity of proteins and sugars displayed on Gram-negative bacteria are potential targets for antibodies. After antibodies bind their target, they can induce effector functions that range from neutralisation of the bacteria and activation of immune cells to the activation of the complement system^3^. Activation of the complement system on Gram-negative bacteria results in the attraction of phagocytes, opsonisation, and the direct killing of the bacteria via the formation of membrane attack complex (MAC) pores^4^. These MAC pores damage the bacterial outer membrane, leading to inner membrane damage and subsequent cell death. MAC pores also enable other immune components or antibiotics to pass the outer membrane and reach their inner membrane- or peptidoglycan-associated targets^5–7^. Complement activation is therefore an important effector function of antibodies directed against Gram-negative bacteria. However, our understanding of what renders antibodies efficacious in inducing effector functions on bacteria is limited.

In humans, antibodies occur in five different isotypes: IgG, IgM, IgA, IgE, and IgD^8^. Antibodies are proteins that all share a Y-shaped structure with two functional domains. The Y-stem is the crystallisable fragment (Fc) and the two arms are the antigen-binding fragments (Fab), which are all linked together with a flexible hinge region. Antibody isotypes differ in their composition of the basic structure and Fc structures and therefore vary in molecular weight and function. IgG has a molecular weight of 150 kDa, is the most prevalent isotype in human serum, and occurs in four subclasses, IgG1-4. IgM is the first isotype to be produced during an infection, mainly occurs in pentameric form, and has a molecular weight of approximately 1000 kDa^9–11^. Of the five antibody isotypes, only IgG1-3 and IgM can activate the complement system via the classical pathway (CP). The CP initiates when the large C1q protein with six collagen arms binds to the Fc domains of an antibody-antigen complex. After C1q binds to antibodies, the C1r and C1s of the C1-complex activate and convert C4 and C2 into a C3-convertase (C4bC2b)^12^. This C3-convertase then converts C3 and deposits C3b on the bacterial surface. At high C3b density, C3 convertases switch substrate specificity to form C5 convertases that cleave C5 into C5a and C5b. C5b subsequently interacts with C6, C7, C8 and multiple copies of C9 to form MAC pores (C5b-9) that can directly kill Gram-negative bacteria^5,13,14^.

In order for IgG to activate the complement system via the CP, six IgG molecules need to bind the surface and assemble in a hexameric structure to enable C1q to bind, as the affinity of C1q to a single IgG is relatively low^15–19^. Recent studies with monoclonal antibodies have shed light on the molecular dynamics of IgG hexamerisation and subsequent complement activation^17,20^. The importance of IgG hexamerisation for complement activation on bacteria is highlighted by engineering strategies improving hexamerisation that in turn enhance complement activation on bacteria^21–26^. In contrast to IgG, IgM has a multimeric structure and when it binds an antigen, it can directly bind C1q. Multiple lines of evidence have suggested that IgM is a more potent complement activator than IgG^16,27–34^. However, the comparison between the complement-activating capacity of IgM and IgG is mainly based on studies with polyclonal or chimeric IgM. Due to the size and multimeric structure of IgM, it has been challenging to produce recombinant monoclonal IgM. Recent advances now enable the production of recombinant monoclonal IgM which allows us to produce IgG and IgM molecules with the same target^35^. Previously we have studied the mechanisms of MAC-mediated killing on *Escherichia coli* as a model for Gram-negative bacteria^13,36,37^. However, the limited number of well-defined monoclonal antibody sequences that target *E. coli* structures hampers the study of how efficiently different antibody isotypes and subclasses activate the complement system on these bacterial membranes.

Here, we set up an artificial antigen–antibody system in *E. coli* MG1655 using StrepTagII as an antigen incorporated into outer membrane protein X (OmpX)^38,39^. This enabled us to directly compare the binding and complement activation capabilities of monoclonal anti-StrepTagII IgG1 and pentameric IgM. We used this in combination with a purified CP complement assay to solely compare the effect of the antibody isotypes in the absence of other serum components. Using these tools, we show IgM’s enhanced capacity over IgG1 to induce complement-mediated lysis of *E. coli*. Altogether, this system can provide valuable insight into the molecular dynamics of how (engineered) IgG and IgM activate complement on bacterial membranes.

## Results

### IgM from serum can trigger classical pathway-mediated killing of *E. coli* MG1655

**Figure 1:**
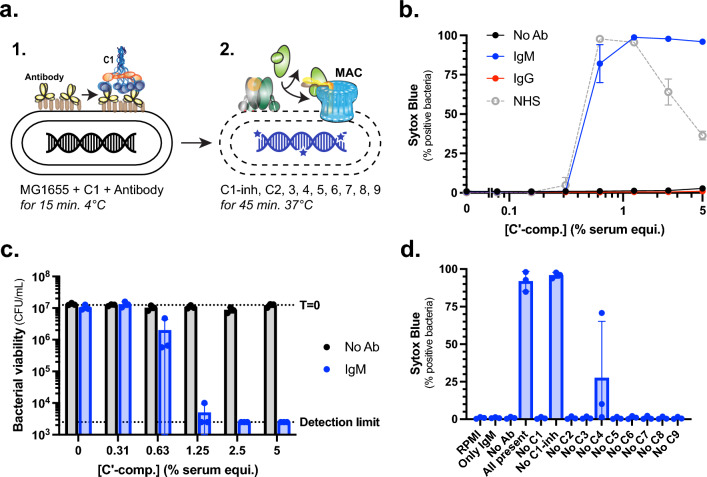
IgM from serum can trigger classical pathway-mediated killing of *E. coli* MG1655. (**a**) Schematic representation of the purified classical pathway assay using the membrane impermeable DNA dye Sytox (blue stars) as readout for inner membrane damage. In the first step, bacteria are mixed with C1-complex, and antibodies for 15 min at 4 °C. In the second step that follows without washing, C1-inhibitor and C2–9 are added for 45 min at 37 °C. (**b**) Bacterial inner membrane damage (percentage Sytox positive) of *E. coli* MG1655 bacteria that were incubated with a concentration range of complement components, with either no antibody added (No Ab), 100 µg/mL polyclonal IgM, or 100 µg/mL polyclonal IgG. The concentrations of complement components correspond to the indicated serum percentages. As a positive control, bacteria were incubated with a concentration range NHS for 45 min at 37 °C. (**c**) Bacterial viability (CFU/mL) of MG1655 after incubation with the purified CP assay with either no antibody added (No Ab) or 100 µg/mL polyclonal IgM. At timepoint 0 min, a sample was taken as is shown as a dotted line with T = 0. The detection limit of the assay is also shown as a dotted line. (**d**) Percentage of the total MG1655 bacterial population that became positive for the DNA dye Sytox blue as measured with flow cytometry. Bacteria were treated either with RPMI, 100 µg/mL polyclonal IgM, 1.25% serum equivalent complement components without antibody, 1.25% serum equivalent with 100 µg/mL IgM (all present), or 1.25% serum equivalent with 100 µg/mL polyclonal IgM without one single indicated complement component. Data represent mean ± SD of three independent experiments (**b**, **c**, and **d**).

To compare antibody isotypes in their ability to induce the killing of *E. coli* MG1655 via the classical pathway (CP), we first considered using serum as a complement source. However, since complement can also be activated via the lectin and alternative pathway in serum, this interferes with pinpointing the effect of antibodies alone. Additionally, serum contains proteins like lysozyme that destroy the bacterial particle after outer membrane permeation by MAC pores, which makes it difficult to solely study the effect of the complement system^7^. To circumvent these limitations, we reconstituted the CP with purified complement proteins (Fig. 1a) by adapting a previously described assay^40^. In this purified CP assay, antibodies and C1-complex are mixed with bacteria for 15 min at 4 °C. After this, C1-inhibitor and the remaining CP complement components (C2—9) are added for 45 min at 37 °C (Fig. 1a). C1-inhibitor is added to prevent unspecific fluid-phase activation of the C1-complex. Previously, we have shown that both outer- and inner-membrane damage are required to achieve a reduction in bacterial viability, which could be measured via the influx of the membrane-impermeable DNA dye Sytox by flow cytometry^6,37^. To induce the CP in this assay, we used polyclonal IgM isolated from healthy donor serum which potently binds *E. coli* MG1655 (Suppl. Figure 1a). This polyclonal IgM induced an efficient influx of Sytox in the bacteria at a concentration of complement components equivalent to 1.25% human serum (Fig. 1b). This corresponds with the concentration where MG1655 is killed in normal human serum (NHS) (Fig. 1b). We did not observe any Sytox influx in the absence of antibodies at any tested concentration of complement components (Fig. 1b). Next, we verified that Sytox influx correlates with a decrease in colony forming unit (CFU) formation. Polyclonal IgM induced > 99% reduction of CFUs at 1.25% serum equivalent complement components, whereas no reduction of CFUs was observed in the absence of antibodies (Fig. 1c). Similarly, NHS induced Sytox influx and a reduction of CFUs at 1.25% (Suppl. Figure 1b, Fig. 1b). The reduction of Sytox signal for higher concentrations of NHS did not correlate with an increased viability, and can most likely be explained by the complete lysis of bacterial particles (Suppl. Figure 1b)^13^. This confirms that Sytox influx is a representative read-out for a reduction of viability of *E. coli* MG1655 bacteria in this purified assay. Additionally, we verified that all CP components and polyclonal IgM are required to achieve Sytox influx (Fig. 1d). Without C1-inhibitor that prevents spontaneous C1 activation in serum, polyclonal IgM still induced bacterial killing, but leaving out any other complement component did not result in Sytox influx (Fig. 1d). Having established an assay to study antibody-induced complement-mediated killing of bacteria, we next aimed to compare IgG and IgM in their ability to do so. Therefore, we also isolated polyclonal IgG from healthy human serum and tested the binding to *E. coli* MG1655. Surprisingly, polyclonal IgG did not bind MG1655 like polyclonal IgM did (Suppl. Figure 1a). The absence of IgG binding correlated with our observations that it could not induce Sytox influx or a reduction of CFUs in the purified assay (Fig. 1b, Suppl. Figure 1b), so we were unable to compare the complement-activating capacities of these IgG and IgM antibodies. In conclusion, we established an experimental procedure to investigate the mechanism of antibody-induced complement-mediated killing of *E. coli* MG1655. However, we cannot use polyclonal antibodies from serum to directly compare complement activation by IgG and IgM.

### Development of a StrepTagII antigen–antibody system to compare IgG- and IgM-mediated complement activation

**Figure 2:**
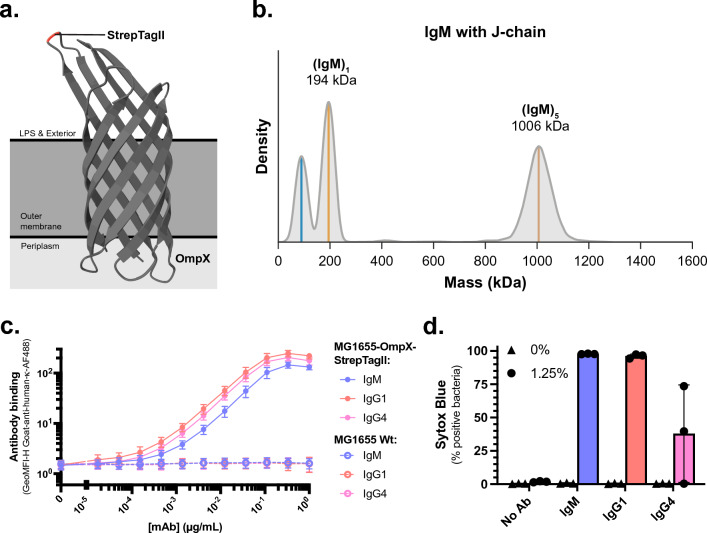
Engineering StrepTagII as antigen and development of anti-StrepTagII monoclonal antibodies. (**a**) Schematic representation of the StrepTagII antigen expressed in loop 2 of outer membrane protein X (OmpX) in the outer membrane of *E. coli*. OmpX structure obtained from the protein data bank^65^. (**b**) Mass photometry density plot of IgM anti-StrepTagII expressed in the presence of the J-chain. Annotated masses correspond to local maxima in density and the peak indicated in blue represents a common contaminant of about 80–90 kDa, possibly a half IgM monomer or single heavy chain. (**c**) Binding of a concentration range of anti-StrepTagII IgM, IgG1, and IgG4 to either MG1655-OmpX-StrepTagII bacteria (solid lines) or MG1655 wildtype bacteria (dotted lines). Antibody binding was detected with Goat-anti-human-kappa-AF488. (**d**) Inner membrane damage (% Sytox positive) of MG1655-OmpX-StrepTagII bacteria treated either with buffer (0%) or with 1.25% serum equivalent complement components with either no antibody (No Ab), 1 µg/mL anti-StrepTagII IgM, anti-StrepTagII IgG1, or anti-StrepTagII IgG4. Samples (**c** and **d**) were measured by flow cytometry and data represent mean ± SD of three independent experiments.

We aimed to directly compare IgG and IgM in their ability to activate the complement system and induce killing of MG1655. However, polyclonal IgG did not bind MG1655 and there are no monoclonal antibodies (mAbs) described to target MG1655 surface structures. Therefore, we required a mAb in combination with an antigen that is expressed on the bacterial surface. For this, we used the anti-StrepTagII mAb, which is described to recognise the eight amino acid peptide StrepTagII with high affinity. To place the antigen on the bacterial surface, we genetically engineered StrepTagII into loop 2 of outer membrane protein X (OmpX) and overexpressed the protein in *E. coli* (Fig. 2a)^39^. We produced anti-StrepTagII as IgG1, IgG4, and pentameric IgM with J-chain. We expressed IgM with J-chain to obtain pentameric IgM because this is the predominant form in human blood^9–11,41,42^. To confirm that we expressed IgM as pentamers, we analysed IgM affinity-purified from the cell culture supernatant by mass photometry (Fig. 2b). IgM co-expression with J-chain yielded ~ 85% (m/m) IgM in pentameric configuration (Fig. 2b). In contrast, if IgM is expressed without J-chain, the resulting sample is highly heterogeneous (containing tetramers, pentamers, and hexamers) (Suppl. Figure 2a and Wörner et al*.*^43^). Therefore, we continued solely with IgM co-expressed with J-chain with subsequent purification by size exclusion chromatography to obtain pure pentameric IgM, hereafter referred to as IgM. To validate that the anti-StrepTagII mAbs specifically recognised the StrepTagII antigen on *E. coli*, we assessed the binding of these mAbs to both MG1655-OmpX-StrepTagII and MG1655 Wt. All three anti-StrepTagII forms, IgG1, IgG4, and IgM, did not bind MG1655 Wt, but bound efficiently to MG1655-OmpX-StrepTagII (Fig. 2c). Subsequently, we validated the ability of the anti-StrepTagII mAbs to activate the complement system on MG1655-OmpX-StrepTagII bacteria. Both IgM and IgG1 could induce complement-mediated killing of MG1655-OmpX-StrepTagII as demonstrated by Sytox influx (Fig. 2d, Suppl. Figure 2b). As expected, no Sytox influx was observed in the absence of complement components or antibodies (Fig. 2d, Suppl. Figure 2b). Whereas IgG4 is not able to potently activate complement^44–46^, it can do so at high antigen and antibody concentrations^47^. Here, IgG4 also induced Sytox influx, but to a lesser degree than IgG1 and IgM (Fig. 2d, Suppl. Figure 2b). In conclusion, we here present an artificial antigen–antibody system in which we can compare IgG and IgM in their ability to activate complement and induce the killing of *E. coli*.

### IgM induces complement activation more efficiently than IgG1

**Figure 3:**
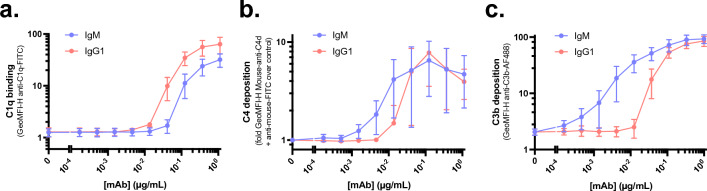
IgM more efficiently induces complement activation than IgG1. MG1655-OmpX-StrepTagII was incubated with a concentration range of anti-StrepTagII IgM and IgG1 and 1.25% serum equivalent complement components. (**a**) C1q binding in the absence of other complement components, by incubating bacteria with antibody and C1-complex for 30 min. (**b**) C4b deposition in the absence of downstream complement components, by incubating bacteria with antibody and C1-complex for 15 min and adding C1-inhibitor and C4 for an additional 15 min. (**c**) C3b deposition with all CP complement components present as described in Fig. 1a. Samples were measured by flow cytometry and data represent mean ± SD of three independent experiments.

Having validated that IgG and IgM anti-StrepTagII bind their target comparably and can both induce inner membrane damage of MG1655-OmpX-StrepTagII bacteria, we aimed to compare the efficiency of IgM and IgG in activating the complement system on these bacteria. First, we compared C1q binding to IgM and IgG1 in the absence of downstream components by detecting C1q. C1q binding to surface-bound IgG1 was slightly more efficient than to surface-bound IgM (Fig. 3a). In contrast, IgM triggered slightly more efficient C4b deposition than IgG1 (in the absence of C3 and other downstream components, Fig. 3b). Lastly, we mixed IgG1 or IgM with all the CP complement components and detected C3b deposition. Herein, IgM more efficiently triggered C3b deposition than IgG1 (Fig. 3c). This difference was more pronounced than for C4b deposition (Fig. 3c). In summary, this data shows that although IgM and IgG1 bind comparably to StrepTagII on the bacterial surface, IgM more efficiently induces complement activation than IgG1.

### IgM triggers complement-mediated killing of MG1655-OmpX-StrepTagII more efficiently than IgG1

**Figure 4:**
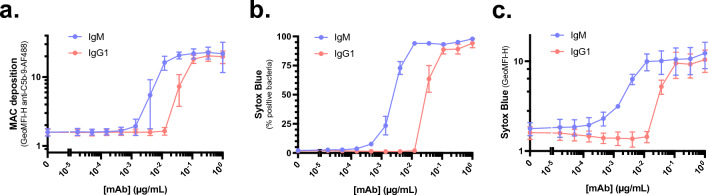
IgM more efficiently triggers complement-mediated killing of MG1655-OmpX-StrepTagII. (**a**) MAC deposition, (**b**) % Sytox positive bacteria, and (**c**) Sytox intensity of MG1655-OmpX-StrepTagII bacteria that were incubated with a concentration range of anti-StrepTagII IgM or IgG1 at a fixed concentration of complement components of 1.25% serum equivalent as described in Fig. 1a. Samples were measured by flow cytometry and data represent mean ± SD of three independent experiments.

In addition to measuring the deposition of complement activation products, we also aimed to compare MAC deposition and killing of bacteria by IgG1 and IgM. To do so, we first compared MAC deposition on MG1655-OmpX-StrepTagII for IgM and IgG1 in the purified CP assay. In line with C3b and C4b deposition measurements, IgM also more efficiently triggered MAC deposition than IgG1 (Fig. 4a). The more efficient MAC deposition by IgM also correlates with increased inner membrane damage and thus killing of MG1655-OmpX-StrepTagII bacteria (Fig. 4b, c). To compare the half maximal effective concentration (EC_50_) of IgG1 and IgM, we fitted four-parameter logistic curves on the Sytox graphs and extracted the EC_50_ values (Suppl. Figure 3a, b, Table 1). Given that IgM and IgG1 significantly differ in their molecular weight (150 kDa for IgG1 and 1000 kDa for pentameric IgM), over six times more IgG1 molecules than IgM are added when comparing the two in µg/mL. Also, since optimal C1q binding occurs when six IgG1 molecules hexamerise^17,48^, we compared C1q binding sites on IgG1 and IgM in a 1:1 ratio in these analyses. However, for the completeness of the analysis, we also analysed the graphs in which the molar concentrations were compared (Suppl. Figure 3c, d). In both conditions, we evaluated the percentage of Sytox-positive bacteria (Suppl. Figure 3a, c) and the Sytox intensity (Suppl. Figure 3b, d, Table 1) and took the average value of these two readouts. When we compare one IgM with six IgG1 molecules, thus in equal C1q binding sites, the EC_50_ of IgM is 11-fold (9–13-fold) lower than the EC_50_ of IgG1. This difference increases to about 74-fold (61–83-fold) when comparing IgM and IgG1 in equimolar ratio (Table 1). Altogether, this data shows that IgM more efficiently drives complement-mediated killing of MG1655-OmpX-StrepTagII bacteria than IgG1.

**Table 1:** The half maximum effective concentration (EC_50_) of IgM is lower than that of IgG1.

	EC_50_ IgG1 (SD)	EC_50_ IgM (SD)	EC_50_ IgG1 / EC_50_ IgM
Sytox influx (%) in µg/mL	3.14E^−2^ (1.61E^−3^)	2.43E^−3^ (9.10E^−5^)	12.9
Sytox influx (FI) in µg/mL	3.93E^−2^ (4.60E^−3^)	4.21E^−3^ (9.88E^−4^)	9.3
Average (µg/ml)			**11.1**
Sytox influx (%) in M	2.09E^−10^ (1.07E^−11^)	2.43E^−12^ (9.10E^−14^)	86.0
Sytox influx (FI) in M	2.62E^−10^ (3.07E^−11^)	4.21E^−12^ (9.89E^−13^)	62.3
Average (M)			**74.2**

The EC_50_ values extracted from the four-parameter logistic curves fitted to the percentage of Sytox positive (%) bacteria and the GeoMFI-H Sytox data (FI). The upper rows are values for concentrations in µg/mL and lower rows are concentrations transformed to molar (M). For each extracted EC_50_ value, the standard deviation (SD) is provided between brackets. The right column shows the EC_50_ value for IgG1 divided over the EC_50_ value for IgM to obtain a ratio for the difference in EC_50_. The average of the values extracted from the % Sytox positive and GeoMFI-H Sytox were calculated and depicted in bold.

### Pre-formed hexameric IgG1 can achieve killing of MG1655-OmpX-StrepTagII as efficiently as IgM

**Figure 5:**
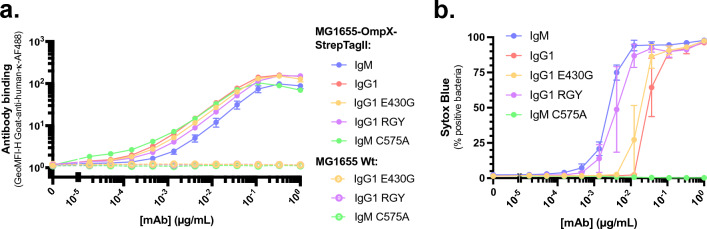
Pre-formed hexameric IgG1 can achieve killing of MG1655-OmpX-StrepTagII as efficient as IgM. (**a**) Binding of a concentration range of anti-StrepTagII IgG1, IgG1 E430G, IgG1 RGY, IgM, and IgM C575A to either MG1655-OmpX-StrepTagII bacteria (solid lines) or for MG1655 Wt bacteria (dotted lines). (**b**) Complement-mediated killing (% Sytox positive) of MG1655-OmpX-StrepTagII after exposure to a concentration range of anti-StrepTagII IgG1, IgG1 E430G, IgG1 RGY, IgM, and IgM C575A at a fixed concentration of 1.25% serum equivalent complement components. Samples were measured by flow cytometry and data represent mean ± SD of three independent experiments.

Previously it was shown that the introduction of specific mutations in the Fc domain of IgGs (E430G (Glu^430^ → Gly) or E345K (Glu^345^ → Lys)) can enhance hexamerisation of target-bound antibodies^17,48^. On several bacterial species (*Neisseria gonorrhea*^24,25^, *Staphylococcus epidermidis*^26^, and *Streptococcus pneumoniae*^22^), these single amino acid mutations were shown to enhance C1q recruitment and downstream complement effector functions^21^. Therefore, we produced anti-StrepTagII IgG1s with the E430G single mutation. In addition, we generated a triple mutant (E345R (Glu^345^ → Arg), E430G (Glu^430^ → Gly), S440Y (Ser^440^ → Tyr), denoted as RGY), because of its ability to form IgG1 hexamers in solution without binding an antigen^18,48^. As counterpart for this pre-assembled hexamer, we produced IgM with the C575A (Cys^575^ → Ala) mutation that prevents IgM from forming pentamers and thus IgM exists as monomers in solution^49,50^. First, we analysed the antibodies with mass photometry to assess their conformation^51^. IgG1 E430G was present in solution as monomers of 150 kDa like wild-type IgG1 (Suppl. Figure 4a, b). IgG1 RGY was found both to form 150 kDa monomers, as well as hexamers thereof (Suppl. Figure 4c). The observed mass of these hexamers is slightly higher than the expected 900 kDa, which is in line with previous findings showing that mass photometry can overestimate the weight of IgG hexamers^51^. IgM C575A was found to form 180 kDa monomers (Suppl. Figure 4d). Next, we validated that the engineered anti-StrepTagII IgGs and IgM C575A bound similar to the MG1655-OmpX-StrepTagII as the wild-type IgG1 and IgM (Fig. 5a). Then, we compared the ability of the engineered IgGs and IgM to induce complement-mediated killing in the purified CP assay. To our surprise, we observed no difference in MAC-mediated killing of MG1655-OmpX-StrepTagII between IgG1-E430G and IgG1-wt (Fig. 5b). In contrast, the IgG1-RGY pre-assembled hexamers showed improved complement-mediated killing over IgG1-wt and induced killing as efficiently as IgM (Fig. 5b). Additionally, whereas we expected IgM C575A monomers to behave similar to IgG1, they did not induce any Sytox influx at any of the measured concentrations (Fig. 5b). The absence of Sytox influx could be attributed to the inability of IgM C575A to bind C1q or trigger C3b-deposition (Suppl. Figure 5a, b). Lastly, we compared the EC_50_ values of IgG1 E430G and IgG1 RGY to IgG1 in molar concentrations to compare equal C1q binding sites like we did for IgM (Suppl. Figure 6a, b, Table 2). The EC_50_ of IgG1 E430G was twofold lower than the one of IgG1, whereas the EC_50_ of IgG1 RGY was 56-fold (48–63-fold) lower than the one of IgG1 (Table 2). Together, this data indicates that preassembled IgG hexamers and IgM pentamers most effectively drive MAC-mediated killing of MG1655-OmpX-StrepTagII.

**Table 2:** The half maximum effective concentration (EC_50_) of engineered IgG molecules compared to IgG1.

	EC_50_ IgG1 (SD)	EC_50_ E430G (SD)	EC_50_ RGY (SD)	EC_50_ ratio
Sytox influx (%) in M	2.09E^−10^ (1.41E^−11^)	1.13E^−10^ (1.02E^−11^)		1.9
Sytox influx (FI) in M	4.53E^−10^ (1.15E^−10^)	1.71E^−10^ (2.60E^−11^)		2.6
Average (IgG1/E430G)				**2.3**
Sytox influx (%) in M	2.09E^−10^ (1.41E^−11^)		4.34E^−12^ (4.57E^−13^)	48.1
Sytox influx (FI) in M	4.53E^−10^ (1.15E^−10^)		7.16E^−12^ (1.49E^−12^)	63.3
Average (IgG1/RGY)				**55.7**

The EC_50_ values extracted from the four-parameter logistic curves fitted to the percentage of Sytox positive (%) bacteria and the GeoMFI-H Sytox data (FI). For each extracted EC_50_ value, the standard deviation (SD) is provided between brackets. The right column shows the EC_50_ value for IgG1 divided over the EC_50_ value for either IgG1 E430G or IgG1 RGY to obtain a ratio for the difference in EC_50_. The average of the values extracted from the % Sytox positive and GeoMFI-H Sytox were calculated and depicted in bold.

## Discussion

Our understanding of which antigen–antibody combinations most potently trigger complement-mediated killing of Gram-negative bacteria is limited by the lack of well-defined antibody-antigen combinations. Here we aimed to compare IgG and IgM in their ability to activate the complement system on *E. coli*. First, we analysed antibody binding using serum from healthy individuals as an antibody source. Surprisingly, we detected ample binding of IgM to *E. coli* MG1655, but we could not detect any functional, surface-bound IgG, so we were unable to use this antibody source to compare IgG and IgM. Furthermore, IgM and IgG from serum are polyclonal and the corresponding antigens are unknown. Thus, even if IgG from serum bound the bacteria, the targets of IgM and IgG could be different which renders it impossible to compare the isotypes directly. Therefore, we set up an artificial antigen–antibody system on *E. coli* to compare the complement-activating capacities of monoclonal IgM and IgG*.* We expressed the eight amino acid peptide StrepTagII in loop 2 of OmpX as a surface-expressed antigen and produced anti-StrepTagII mAbs in different isotypes^38,39^. We tested the activity of these antibodies in a purified classical pathway system, where we mixed our bacteria with purified complement components. This eliminates several problems that arise when using serum as a complement source to measure antibody activity. In full serum, immune components such as lysozyme and phospholipase II act on the bacterial peptidoglycan layer and the bacterial inner membrane when MAC pores are formed in the outer membrane^7^. This makes it difficult to pinpoint which effects are caused by antibodies and the complement system. Additionally, complement activation by the lectin and alternative pathway in serum makes it challenging to solely study antibody-mediated complement activation. Here we show that antibodies and complement are sufficient to induce inner membrane damage. Despite the advantages of excluding these serum factors to pinpoint the role of antibodies alone, future studies are needed to address whether the alternative- and lectin pathway and other serum components could accelerate the effect seen in this purified CP assay. All in all, this assay allowed us to directly compare different (engineered) antibodies in their capacity to trigger complement-mediated killing of *E. coli*.

Using the experimental setups described above, we showed that pentameric IgM is an about 70-fold more efficient complement activator and inducer of MAC-dependent bacterial killing than IgG1. Anti-StrepTagII antibody has been described to have a high affinity Fab for StrepTagII, and the affinity might be an important determinant for this effector function of antibodies. In line with our results, high affinity O-antigen targeting IgMs have previously been described to be 20–140-fold more vibriocidal than IgG on *Vibrio cholerae*^52^. In contrast, another study with chimeric antibodies has shown that IgMs exhibited similar serum bactericidal activity as IgG1 when targeting porin A in the outer membrane of *Neisseria meningitidis*^53^. However, these chimeric IgG and IgM antibodies bound at EC_50_ values of about 2.5 µg/mL, indicating they have less affinity for their antigen than the mAbs used here. The differences of how IgG1 and IgM compare in activating the complement system might therefore be (partly) explained by the affinity of the Fab for the antigen. However, several other factors might also play a role, such as antigen location or antigen density. Our results are obtained with the StrepTagII antigen as part of overexpressed OmpX. Recent studies have demonstrated that on *E. coli* MG1655, there are patches rich in Omps and patches rich in lipopolysaccharides (LPS)^54^. These patches differ in membrane lipid composition which could interfere with MAC-insertion into the membrane. Therefore, the location of the antigen of the antibody could have implications for the capacity of MAC-insertion and thereby complement-mediated killing. Since different antibodies binding to the LPS are described to both activate and inhibit complement-mediated killing of Gram-negative bacteria^52,55^, we hypothesise that both the location, but also the distance of the antigen to the membrane might be an important determinant for whether an antibody can induce complement-mediated killing or not. On single membrane particles, an epitope further away from the membrane was demonstrated to induce less complement-dependent lysis than an epitope closer to the membrane^56^. We hypothesise that if complement activation by antibodies takes place too far from the membrane, the direct local anchoring of the MAC into the membrane might be impaired^36^. The importance of antigen density was previously highlighted by the fact that hexameric IgM has superior complement activating capabilities over pentameric IgM and IgG1 if the antigen density is low, whereas at high densities they all three matched^57^. Our results are obtained with an overexpressed antigen and the observed difference in complement activation between IgM and IgG1 should be investigated for this type of antigen at different expression levels and thus antigen densities. To study the influence of antigen density, the expression of OmpX-StrepTagII could be varied by using a variable inducible expression vector. Furthermore, the antigen location could be varied by attaching StrepTagII to the LPS of *E. coli* via click chemistry, by clicking DBCO-StrepTagII to KDO-azide which can be incorporated into the LPS^58^. Lastly, to alter the distance of StrepTagII to the membrane, we could make use of flexible linkers to attach the antigen to the LPS.

In this study, we show that pentameric IgM is more efficient than IgG1 in driving complement-mediated killing of *E. coli*. RGY mutations in IgG1, that trigger the formation of pre-assembled hexamers in solution^18^, enhance the activity of IgG1 to induce killing of MG1655 as efficiently as pentameric IgM. In contrast, the monomeric variant of IgM could not induce killing of MG1655, most likely due to its inability to bind C1q. In addition, we showed that mutations in IgG1 that enhance on-target hexamerisation^48^ could not overcome the difference between IgG1 and IgM. This contradicts our previous findings where these enhanced on-target hexamerisation forming IgGs significantly improved complement activation on *Streptococcus pneumoniae*^21,22^. The here-presented results were obtained with the Gram-negative bacterium *E. coli* in which the antigen type, density, and location differs from the Gram-positive *S. pneumoniae* which could explain the observed differences.

In conclusion, we here present an antigen–antibody system which allows us to directly compare the capacity of (engineered) antibodies to activate complement on live bacteria. This system could also be used to study the effect of antibody isotype on other antibody effector functions like phagocytosis. We also employed a purified complement assay to compare the effect of antibodies alone in their ability to induce complement-mediated killing. In the future, this assay could be used to study patient derived antibodies to understand their immune response during or after bacterial infections. Since the broad range of effector functions of antibodies render them an attractive alternative to antibiotics to treat or prevent bacterial infections^3,59,60^, studying the relationship between antigens and antibodies is a key prerequisite to guide the design of new therapeutic antibodies.

## Materials and methods

### Serum and complement proteins

Normal human serum (NHS) was obtained from a pool of healthy donors as previously described^61^. Complement components C1-complex, C1-inhibitor, C2, C4, and C8 were obtained from Complement Technologies. His-tagged C5, C6, and C7 were expressed in HEK293E cells at U-Protein Express as described previously^36^. C9-3xGGGGS-LPETG-6xHis was recombinantly expressed in Expi293F cells^13^. C3 was isolated from plasma as previously described^61^.

### Antibodies

Polyclonal IgM was isolated from NHS over a POROS™ CaptureSelect™ IgM Affinity Matrix (ThermoScientific) using an ÄKTA Pure (GE Healthcare). The IgM was dialysed overnight against PBS with 0.5 M NaCl (PBS500). The dialysed IgM was run over a Superose 6 Increase 10/300 GL column (Cytiva) and fractions pooled to increase and assess purity to ≥ 95%. Polyclonal IgG was isolated from NHS using a HiTrap Protein G High Performance column (GE Healthcare). After capture, IgG was eluted according to the manufacturer’s instructions and dialysed overnight against PBS. For both polyclonal IgM and IgG a working stock was stored at 4 °C and a long-term storage stock at −80 °C.

The heavy and light chain variable region (VH and VL) amino acid sequences of monoclonal anti-StrepTagII were from patent (WO 2015/067768 A1, 2015). IgG1, IgG4, IgG1 E430G, and IgG1 RGY were produced as described previously^21,23,62^. For monoclonal IgM production, gBlocks containing codon optimised VH and VL sequences with an upstream KOZAK and HAVT20 signal peptide were cloned into adapted pcDNA34 vectors (ThermoFisher Scientific), upstream the IgM heavy and kappa light chain constant regions, respectively, using Gibson assembly (New England Biolabs), as described previously^22^. BamHI and BsiWI were used as the 3’ cloning sites for the VH and VL respectively, in order to preserve the immunoglobulin heavy and kappa light chain amino acid sequence. A plasmid coding for J-chain expression with the following sequence: QEDERIVLVD NKCKCARITS RIIRSSEDPN EDIVERNIRI IVPLNNRENI SDPTSPLRTR FVYHLSDLCK KCDPTEVELD NQIVTATQSN ICDEDSATET CYTYDRNKCY TAVVPLVYGG ETKMVETALT PDACYPD was kindly provided by Theo Rispens. For the production of monomeric IgM, a C575A mutation was introduced in the IgM heavy chain by PCR and cloned in an adapted pcDNA34 vector. Subsequent cloning of the VH region was performed as described above. After verification of the correct sequence, the plasmids were used to transfect EXPI293F cells (ThermoFisher Scientific). EXPI293F cells were grown in EXPI293 medium in culture filter cap Erlenmeyer bottles (Corning) on a rotation platform (125 rotations/min) at 37 °C, 8% CO_2_. One day before transfection, cells were diluted to 2 × 10^6^ cells/mL. The next day, cells were diluted to 2 × 10^6^ cells/mL with SFM4Transfx-293 medium containing UltraGlutamine I (VWR International) prior to transfection using PEI (Polyethylenimine HCl MAX; Polysciences). For transfection, 1 µg DNA/mL cells (ratio of heavy and light chain plasmids is 2:3 for IgM and 1:3 for monomeric IgM) was added to OPTIMEM (1:10 of total volume; Gibco) and gently mixed. For expressions of IgM containing the J-chain, the J-chain plasmid was used as 50% of total plasmid. After adding PEI (1 µg/mL; ratio PEI to DNA is 5:1), the mixture was incubated at room temperature for 20 min and then added dropwise to the cells while manually rotating the Erlenmeyer culture bottle. After 4 to 5 h, 1 mM Valproic Acid (Sigma) was added to the transfected cells. After 5 days of expression, the cell supernatant was collected by centrifugation and filtration (0.45 µm) and subsequently dialysed against PBS. After dialysis, extra NaCl was added to the IgM and monomeric IgM preparation to a final of 0.5 M, before application to a POROS™ CaptureSelect™ IgM Affinity matrix column. IgM and monomeric IgM were eluted with 0.1 M Glycine–HCL pH 3.0. on the ÄKTA Pure. 0.5 M NaCl was added to the pooled fractions, which were neutralised with 1 M Tris pH 7.5. Both IgM isolates were dialysed against PBS500. Finally, IgM with or without J-chain and monomeric IgM were isolated on a Superose 6 Increase 10/300 GL to ≥ 95% purity. For all mAbs, a working stock was stored at 4 °C and a long-term storage stock at −80 °C.

### Bacterial strains and culture

All experiments were performed with *E. coli* MG1655 wildtype or MG1655-OmpX-StrepTagII as specified with the results. To obtain MG1655-OmpX-StrepTagII, MG1655 was transformed with a modified pULTRA vector containing a kanamycin resistance cassette and the following sequence for OmpX-StrepTagII with StrepTagII underlined: MKKIACLSAL AAVLAFTAGT SVAATSTVTG GYAQSDAQGQ MNKMGGFNLK YRYEEDNSPL GVIGSFTYTE KSRTASGQSG QWSHPQFEKG GSSGDYNKNQ YYGITAGPAY RINDWASIYG VVGVGYGKFQ TTEYPTYKHD TSDYGFSYGA GLQFNPMENV ALDFSYEQSR IRSVDVGTWI AGVGYRF.

For all experiments, a single bacterial colony was picked from Lysogeny Broth (LB) agar plate (MG1655-OmpX-StrepTagII with 50 µg/mL kanamycin) and grown overnight in LB medium (for MG1655-OmpX-StrepTagII with 50 µg/mL kanamycin) whilst shaking at 37 °C. Bacteria were diluted 1/30 and 1/100 in fresh LB medium and grown until mid-log phase (OD_600_ 0.4–0.6) shaking at 37 °C. Once at mid-log phase, bacteria were pelleted by centrifugation and resuspended in RPMI 1640 medium (Thermofisher/Gibco) containing 0.05% human serum albumin (RPMI-HSA) to OD_600_ = 1 (~ 1 * 10^9^ bacteria/mL).

### Antibody binding assay

Bacteria were grown as described above and incubated with an antibody concentration as denoted per experiment at a bacterial concentration of OD_600_ = 0.0125 in RPMI-HSA for 30 min at 4 °C under shaking conditions. Subsequently, for monoclonal antibodies, the bacteria were washed with RPMI-HSA, pelleted, and resuspended in 3 µg/mL Goat-anti-human-kappa-AF488 (Southern Biotech, 2060-30) detection antibody for 30 min at 4 °C whilst shaking. In case of polyclonal antibodies, the detection antibodies were 3 µg/mL of anti-IgM-PE/Dazzle594 (Biolegend, 314530) or anti-IgG-FITC (Southern Biotech, 2040-02). After incubation, the bacteria were washed in RPMI-HSA, pelleted, and resuspended in RPMI-HSA. The antibody binding to bacteria was measured by flow cytometry on a MACSQuant VYB (Miltenyi Biotec).

### Purified classical pathway assay

Purified complement components were used in concentrations in physiological ratio as they occur in (diluted) serum, denoted as percentage serum equivalent^40,63^. Per component in 100% serum: C1-complex 135 µg/mL; C1-inhibitor 180 µg/mL; C2 20 µg/mL; C3 1250 µg/mL; C4 400 µg/mL; C5 70 µg/mL; C6 64 µg/mL; C7 56 µg/mL; C8 55 µg/mL; and C9 60 µg/mL. As first step of the purified classical pathway assay, bacteria (OD_600_ = 0.025) were incubated with an indicated concentration of antibody and C1-complex for 15 min, shaking at 4 °C. Subsequently, the remaining components C1-inhibitor, C2, C3, C4, C5, C6, C7, C8, and C9 were added at twice the concentration of the desired final concentration in a volume that is equivalent of that of the first step, and incubated for 45 min shaking at 37 °C. This renders the final concentration of C1-complex half that of the other complement components and the final bacteria concentration OD_600_ = 0.0125.

### Bacterial viability and complement deposition

Bacterial viability was measured in either an indicated percentage of normal human serum (NHS) for 45 min at 37 °C or the purified CP assay as described above. To measure Sytox influx, Sytox Blue (Thermofisher) was added during the incubation at a final concentration of 1 µM. After the incubation, the samples were diluted in cold RPMI-HSA and immediately measured by flow cytometry on a MACSQuant VYB. To analyse C3b and MAC deposition, the samples were treated similarly as for Sytox measurements and after a total incubation of 1 h, the bacteria were washed with RPMI-HSA, resuspended in 1 µg/mL monoclonal mouse anti-C3b (clone bH6^64^), randomly labelled with NHS-Alexa Fluor 488 (AF488, Thermofisher Scientific) as done previously^13^, or monoclonal anti-C5b-9-AF488 (clone aE11) and incubated for 30 min at 4 °C whilst shaking.

For analysis of C1q binding, bacteria were incubated with a concentration range of specified antibodies and C1-complex at the specified concentrations for 30 min at 4 °C whilst shaking. Afterwards, the bacteria were washed with RPMI-HSA, resuspended in 3 µg/mL polyclonal rabbit-anti-human-C1q-FITC (Dako, F0254) and incubated for 30 min at 4 °C whilst shaking.

To analyse C4b deposition, bacteria were incubated with the specified antibodies in the first step of the purified CP assay as described above. After 15 min, C1-inhibitor and C4 were added twice concentrated of the desired concentration in a volume that is equivalent of that of the first step, and incubated for 15 min shaking at 37 °C. Afterwards, the bacteria were washed with RPMI-HSA, resuspended in 3 µg/mL murine monoclonal anti-human C4d (Quidel, A213) and incubated for 30 min at 4 °C whilst shaking. Again, the bacteria were washed with RPMI-HSA, resuspended in 3 µg/mL Goat F(ab’)2-anti-mouse-IgG-FITC (Southern Biotech, 1030-02) and incubated for 30 min at 4 °C whilst shaking. After incubation with a detection antibody, the bacteria were washed in RPMI-HSA, pelleted, and resuspended in cold RPMI-HSA. The detection antibody binding to bacteria was measured by flow cytometry on a MACSQuant VYB.

### Mass photometry

Mass photometry experiments were performed on a Refeyn OneMP mass photometer (Refeyn) similar to described before^51^. Microscope coverslips (24 mm × 50 mm; Marienfeld) were prepared by sequential cleaning in sonication baths of isopropanol and then MilliQ water (2x), followed by placement of a CultureWell gasket (Grace Biolabs). About 15 µL of PBS was placed in a well for focusing, after which about 3 µL of diluted sample was mixed in. Measurement concentrations were typically around 5–20 nM. Measurements were recorded using medium field-of-view settings for 120 s. An in-house calibration mix consisting of IgG4Δhinge-L368A (73 kDa), IgG1-Campath (149 kDa), apoferritin (483 kDa), and GroEL (800 kDa) was used. Recordings were processed in DiscoverMP (Refeyn), from which also the relative abundances of protein complexes were obtained. Further data analysis and plotting were performed in Jupyter Notebook using an in-house Python library.

### Data analysis

Flow cytometry data was analysed using FlowJo V10 and graphs including standard deviation were constructed using Graphpad Prism 9. Mass photometry data was processed in DiscoverMP (Refeyn) and analysed using an in-house python library. To obtain the fitted curves and half-maximum values for these, the nonlinear analysis function ‘Sigmoidal, 4PL, X is concentration’ function of Graphpad Prism 9 was used.

### Ethics statement

Human blood was isolated after informed consent was obtained from all subjects in accordance with the Declaration of Helsinki. Approval was obtained from the medical ethics committee of the UMC Utrecht, The Netherlands.

### Supplementary Information

Supplementary Figures.

## Data Availability

All data generated or analysed during this study is included in the published article and its Supplementary Information.
