# Supplementary material for: Artificial surface labelling of Escherichia coli with StrepTagII antigen to study how monoclonal antibodies drive complement-mediated killing
Source: Sci Rep. 2023 Nov 1;13:18836. doi: 10.1038/s41598-023-46026-x (PMC10620216; doi:10.1038/s41598-023-46026-x)
Supplement: Supplementary file 1 — Supplementary Figures. [file 41598_2023_46026_MOESM1_ESM.pdf]

Supplementary material

Artificial surface labelling of *Escherichia coli* with StrepTagII antigen to study how monoclonal antibodies drive complement-mediated killing

Remy M. Muts[1], Maurits A. den Boer[2,3], Bart W. Bardoel[1], Piet C. Aerts[1], Carla J.C. de Haas[1], Albert J.R. Heck[2,3], Suzan H.M. Rooijakkers[1], Dani A.C. Heesterbeek[1]*

[1]*Department of Medical Microbiology, University Medical Center Utrecht, 3584 CX Utrecht, The Netherlands*
[2]*Biomolecular Mass Spectrometry and Proteomics, Bijvoet Center for Biomolecular Research and Utrecht Institute of Pharmaceutical Sciences, Utrecht University, 3584 CH Utrecht, The Netherlands*
[3]*Netherlands Proteomic Center, 3584 CH Utrecht, The Netherlands*

*Corresponding Author: Dani Heesterbeek, Email: D.A.C.Heesterbeek-2@umcutrecht.nl

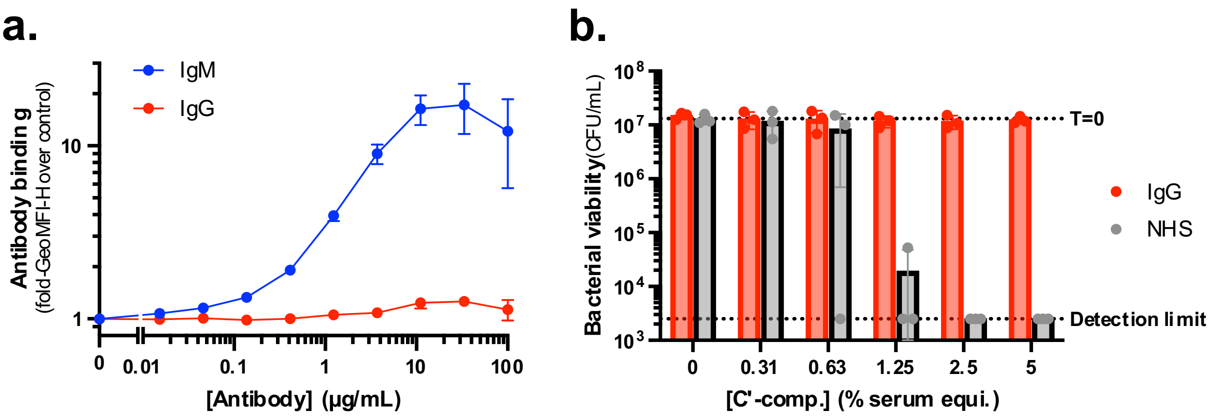

Supplemental figure 1. Polyclonal IgM, but not IgG, binds to and triggers killing of E. coli MG1655.
a) Binding of a concentration range of polyclonal IgM or IgG to MG1655 bacteria detected with anti-IgM-PE/Dazzle594 or anti-IgG-FITC respectively. Geometric mean fluorescence intensity height (GeoMFI-H) signals were normalised to the detection antibody control. b) Bacterial viability (CFU/mL) of MG1655 after incubation with a concentrations range of NHS for 45 minutes at 37°C or the purified CP assay with 100 µg/mL polyclonal IgG. At timepoint 0 minutes, a sample was taken as is shown as a dotted line with T=0. The detection limit of the assay is also shown as a dotted line. Data represent mean ± SD of three independent experiments.

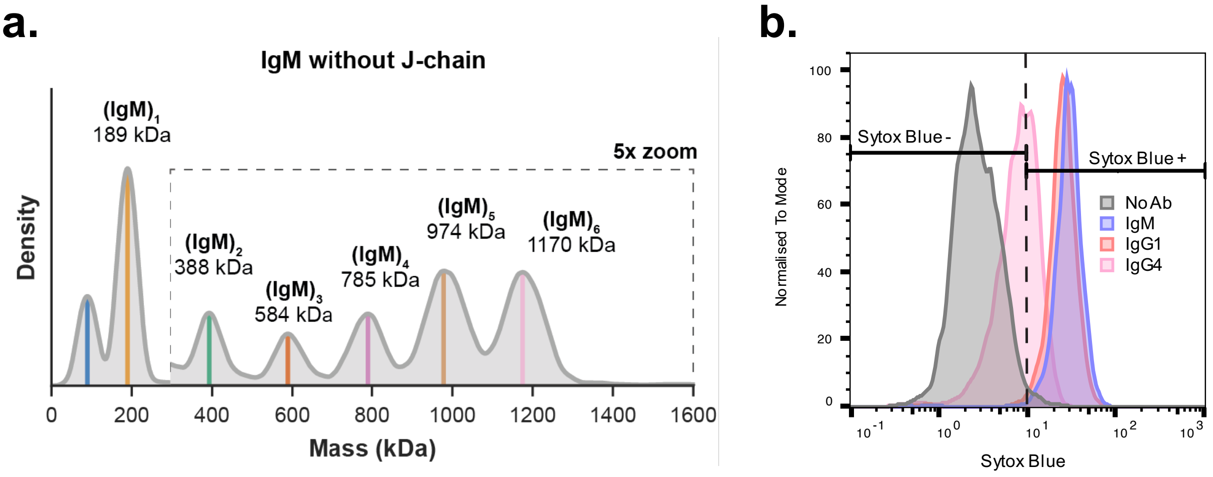

Supplemental figure 2. Development and validation of anti-StrepTagII monoclonal antibodies.
a) Mass photometry density plot of IgM anti-StrepTagII expressed in the absence of the J-chain. Annotated masses correspond to local maxima in density and the peak indicated in blue represents a common contaminant of about 80-90 kDa, possibly a half IgM monomer or single heavy chain. b) Representative histograms of the Sytox blue intensity of MG1655-OmpX-StrepTagII bacteria treated with 1.25% equivalent complement components similar to figure 2d. The presented plots are a representative of three independent experiments.

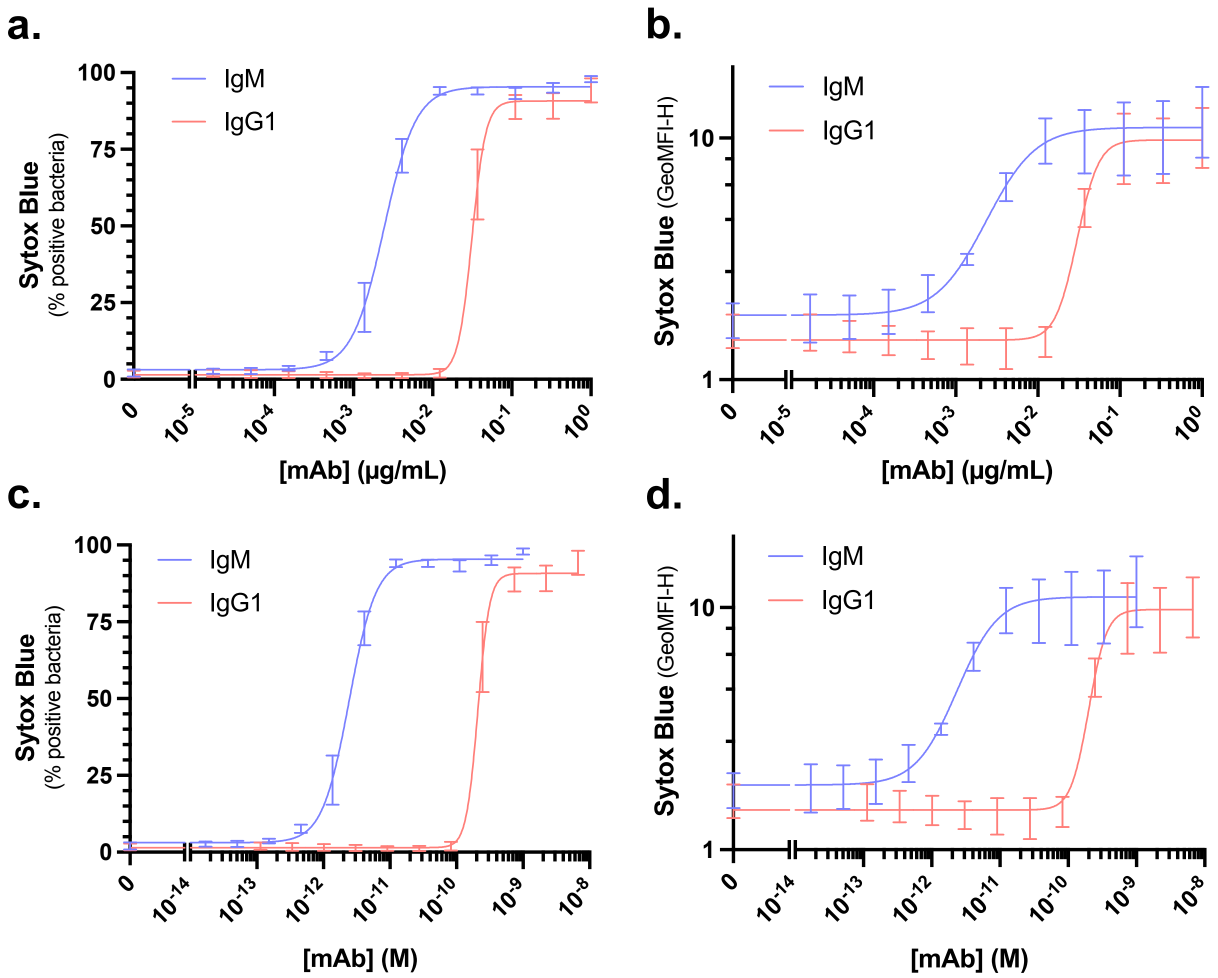

Supplemental figure 3. Fitted curves provide EC_{50} values to calculate the IgG1-IgM difference.
Fitted sigmoidal, 4PL, curves to the Sytox Blue data of a) figure 4b and b) figure 4c. The values in µg/mL on the x-axis are transformed for the molecular weight of IgM (1000 kDa) and IgG1 (150 kDa) to obtain values in Molar. To these transformed graphs from figure 4b and figure 4c new sigmoidal, 4PL, curves are fitted to obtain c) and d) respectively. The fitted curves were used to extract EC_{50} values for IgM and IgG1.

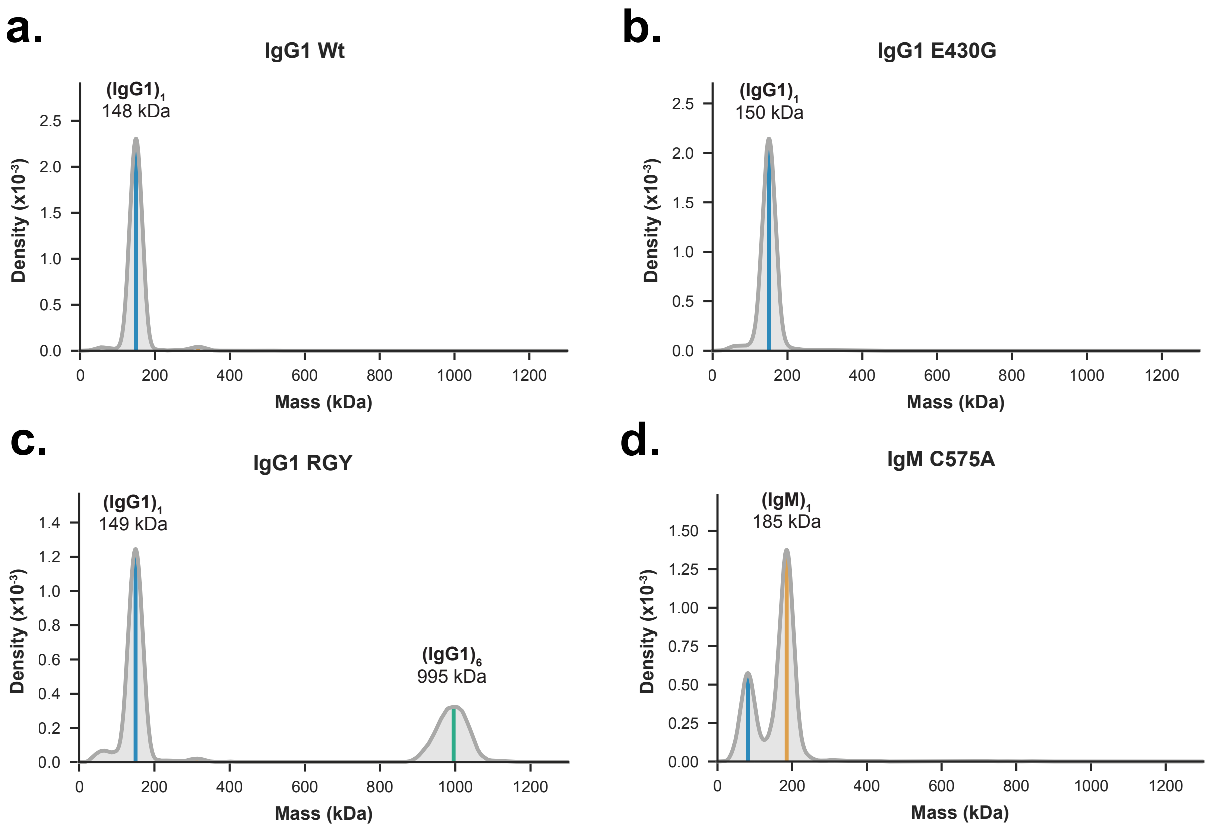

Supplemental figure 4. Validation of anti-StrepTagII monoclonal antibodies.
Mass photometry density plot of anti-StrepTagII a) IgG1, b) IgG1 E430G, c) IgG1 RGY, and d) IgM C575A. Annotated masses correspond to local maxima in density. Annotated masses correspond to local maxima in density. The peak indicated in blue d) represents a common contaminant of about 80-90 kDa, possibly a half IgM monomer or single heavy chain.

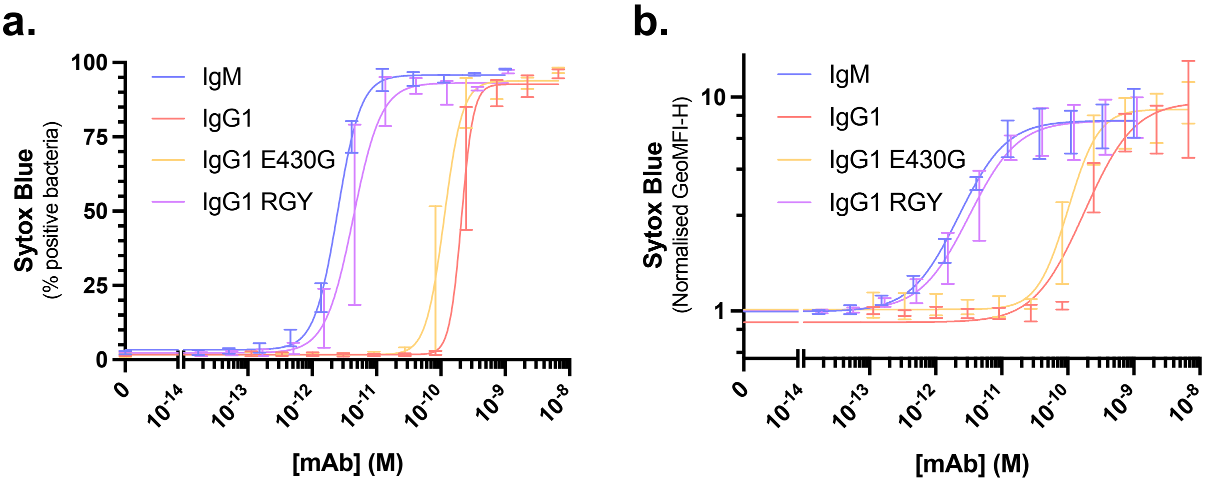

Supplemental figure 5. Fitted curves provide EC_{50} values to calculate the EC_{50} difference with IgG1.
Fitted sigmoidal, 4PL, curves to the Sytox Blue data of a) figure 5b and b) the GeoMFI data corresponding to figure 5b. The values in µg/mL on the x-axis are transformed for the molecular weight of IgM (1000 kDa), IgG1 (150 kDa), IgG1 E430G (150 kDa), and IgG1 RGY (900 kDa) to obtain values in molar. The fitted curves were used to extract EC_{50} values for IgG1, IgG1 E430G and IgG1 RGY displayed in Table 2.

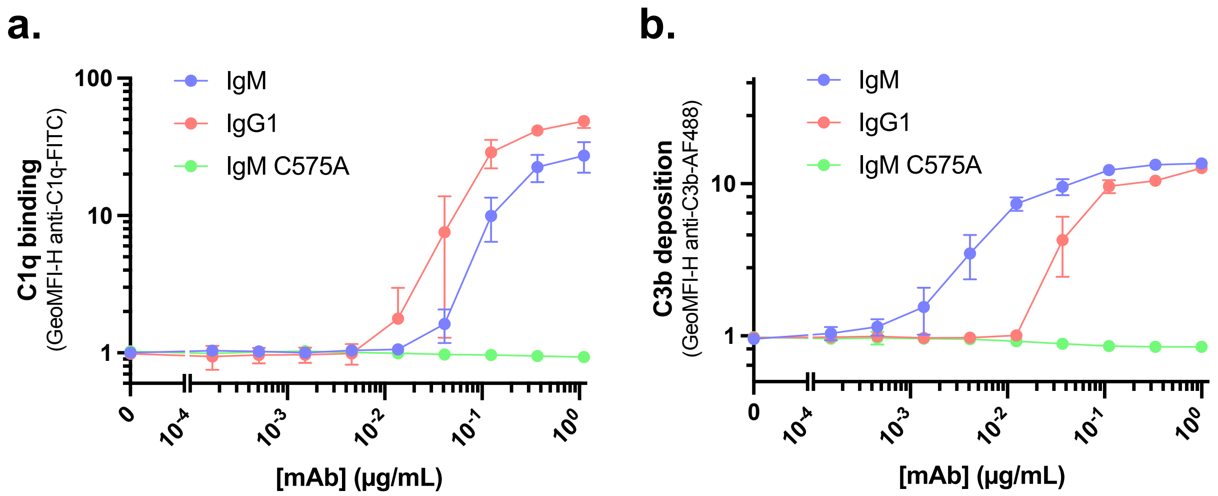

Supplemental figure 6. IgM C575A does not activate the complement system.
MG1655-OmpX-StrepTagII was incubated with a concentration range of anti-StrepTagII IgM, IgM C575A, or IgG1 and 1.25% serum equivalent complement components. a) C1q binding in the absence of other complement components, by incubating bacteria with anti-StrepTagII antibodies and C1-complex for 30 minutes. b) C3b deposition with all CP complement components present as described in figure 1a. Samples were measured by flow cytometry and data represent mean ± SD of three independent experiments.
